# Effects of acute alcohol ingestion on eye movements and cognition: A double-blind, placebo-controlled study

**DOI:** 10.1371/journal.pone.0186061

**Published:** 2017-10-12

**Authors:** Jéssica Bruna Santana Silva, Eva Dias Cristino, Natalia Leandro de Almeida, Paloma Cavalcante Bezerra de Medeiros, Natanael Antonio dos Santos

**Affiliations:** 1 Department of Psychology, Federal University of Paraíba, João Pessoa, Paraíba, Brazil; 2 Department of Medicine, Federal University of Ceará, Sobral, Ceará, Brazil; 3 Department of Psychology, Federal University of Piauí, Parnaíba, Piauí, Brazil; University of Leicester, UNITED KINGDOM

## Abstract

Alcohol is one of the most consumed psychoactive substances in the world, and the negative impact related to alcohol use has become a worldwide public health issue. Alcohol is able to affect diffusely several areas of the Central Nervous System, which could impair visual functions, including eye movements, and cognitive processes. The objective of the present study was to investigate the effects of moderate alcohol intake in eyes movements, as an indicator of cognitive processing underlying the visual search in a the Maze task. We investigated the concentration of 0.08% blood alcohol concentration (BAC), using an intra-subject, double-blind, and placebo-controlled experimental design with a sample size of 20 young adults (11 men and nine women). All volunteers participated in both conditions, i.e., alcohol (0.08%) and placebo (0.00%), in a counterbalanced order. We use the Tobii TX300 eye tracker to evaluate eye movements during completion of Visual Maze Test. The results showed significant differences in the following eye movement patterns: the first fixation latency, number and duration of fixations (mean and total), the number and duration of saccades (mean and total), and the total execution time in the test. In addition, we investigate the areas of interest (AOI), decision points in which the participant must decide which course to follow. We verified that the participants in the alcohol condition had a significantly greater number of fixations in both AOI, in comparison to the placebo condition. Overall, our findings confirm that moderate doses of alcohol can change the eye movements of young adults. These alterations may evidence the influence of alcohol in cognitive processes, such as flexibility, attention, and planning, which are required during resolution of Maze Task.

## Introduction

The consumption of alcoholic beverages is popular worldwide, and it is considered a public health issue due to the serious long-term personal, social, and economic damage [1–3]. In addition to these socioeconomic outcomes, alcohol ingestion may cause varying degrees of physiological losses that may result in changes in the cognitive and behavioral functions as well as visual perception [4–5].

Intoxication due to occasional alcohol ingestion may affect the central nervous system (CNS). The effects may be felt and measured even when alcohol is consumed in light to moderate levels [6]. According to Edward, Marschal and Cook, alcohol acts as CNS depressor, acting in various areas, among which are the reticular formation, the spinal cord, the cerebellum and the cerebral cortex [7]. Although the mechanisms of action for alcohol have not been fully understood, there is evidence that several neurotransmitters are affected by this substance [8–9]. It is possible that alcohol may cause many of its effects through the modulation of the dependent ion-channels associated with the GABAergic (GABA–gamma-aminobutyric acid), glutamatergic (NMDA–N-methyl-D-aspartate), serotoninergic, and dopaminergic neurotransmitters [10–11].

The effects of alcohol on visual functions may be related to a potential reduction in the activity of GABA, which is the main inhibitory neurotransmitter in the brain. One of the examples of evidence that supports this hypothesis shows that the administration of GABA activity enhancers is an efficient method of ceasing alcohol ingestion [12]. In addition, the reduction in the levels of this recept in the brain of alcoholic individuals supports the hypothesis that the interaction between alcohol and GABA is implicated in a dysfunction in the CNS [13–14]. This neurotransmitter is found in different structures involved in visual information processing, such as the ganglion and bipolar retinal cells, the lateral geniculate nucleus (LGN), superior colliculus (SC) and the visual cortex, which are structures that are involved in visual information processing [11–12]. GABA is also involved in the control of ocular movement, the speed which a monkey performs eye movement can be increased or decreased by injection into SC of GABA agonists or antagonists, respectively [15–16].

These effects of alcohol on the CNS may result in alterations in the visual system that are related, for instance, to color perception [6], contrast sensitivity [2], as well as on eye movements [5, 17, 18]. Typical eye movements whilst scanning an image can be classifies as saccades and fixations [19–21]. Saccades are ballistic movements of the eye itself from one point of the visual scene to another, whereas fixations refer to the time between the saccades in which the eye presents minimal movements [21]. Considerable progress has been recorded in the understanding of the brain processes that assist in the eye movement control. The CS of the mesencephalon is closely involved in the eyes movement control, receiving visual information from multiple cortical regions, as the region of the rostral pole of CS is crucially involved in fixation and triggering of the saccade [16].

One of the main functions of eye movements is to align information of potential interest and the fovea, where retinal cones are particularly concentrated, thus selecting information from relevant parts of the visual environment [22]. Based on Hoffman, eye movements are closely related to visual attention; therefore, several studies have used the analysis of saccade target regions to investigate the relation between eye movements and covert attention, suggesting that attention is oriented in the same direction as the saccades [23]. It is generally agreed that visual attention can be determined by two complementary processes that act in the ocular screening of scenes: a bottom-up mechanism, guided by intrinsic stimulus properties that involves perceptive and neurophysiological activity, guiding the attention towards certain stimuli that are physically predominant in relation to others [24]; as well as top-down processes, that require cognitive activity in order to set the goals for a desirable orientation: visual attention is oriented, establishing an inhibitory control of irrelevant stimuli, being influenced by concepts, expectations and visuospatial memory to perform the task [25].

The method of eye movement tracking has been performed to evaluate distinct motor, perceptual and cognitive process under the influence of alcohol. For instance, Abroms, Gottlob, and Fillmore evaluate the effects of moderate alcohol ingestion on inhibitory control (intentional and automatic) over selective attention [17]. In this study, the task performance was tested under three doses of Absolute alcohol, 0.0 (placebo), 0.45, and 0.65 g/kg. The participants were to perform a horizontal saccade in the direction of a target, and selective attention was measured by the fixations and saccadic movements during the performance of the task. The results showed that attention processing dependent on the eye intentional inhibitory control may be susceptible to the harmful effects of a moderate dose of alcohol (for more information on automatic inhibitory control, see Jasinska [26].

Another study conducted by Marinkovic et al. evaluates the effects of a moderate dose of alcohol (0.6 g/kg alcohol for men and 0.55 g/kg for women) on oculomotor system through the inhibition of saccadic responses. To that end, eye tracking was performed during the execution of visually guided saccades into a target and anti-saccades from the same target. The results suggested that alcohol intoxication may impair top–down regulative functions by increased saccades, decreased self-control, that is reduced guided visual behavior toward a goal [5].

Among the possible consequences of alcohol use, Lawrence, Luty, Bogdan, Sahakian and Clark also emphasize that individuals may present impairment in the ability to make decisions [27], a function which involves choosing between two or more competing alternatives, and in which other cognitive processes are involved, such as flexibility, inhibitory control, attention, planning, among others [28]. Although the majority of studies that evaluate the relationship between alcohol intake and decision making using cognitive tests [29–30], eye tracking has been used in the analysis of the ability to choose [31], however there is a scarcity in the literature of studies that survey the effects of alcohol in decision making from parameters of ocular movements.

According to Krajbich, Armel and Rangel, the evidence that helps during a decision, is accumulated during the fixations, indicating that attention plays an active role in the choice process [3, 24]. In addition, it is during the fixations that we plan the orientation and direction of the next saccade, indicating the existence of cognitive processing [32]. From this perspective, the need for a greater number of fixations may indicate a lower efficiency of the information cognitive processing during a visual search [33]. Studies assessing eye movements are considered relevant due to the essential role of the eye movements in acquiring information while processing visual stimuli, allowing us to analyse how visual information is acquired, represented, and stored, providing a direct and instant measurement of cognitive processes, such as attention and decision making [16, 34, 35].

Thus, bearing in mind that the alcohol is able to affect diffusely several areas of the central nervous system, which could impair the cognitive, motor and perceptual skills [7], we hypothesized that young adults under the influence of a moderate dose of alcohol may present subclinical alterations in their eye movements, which may indicate impairment in cognitive processing, compared to the performance of these individuals in the placebo condition [5, 16, 17]. We expect that these changes be characterized by a larger number and duration of fixations, higher latency for the initial fixation and a higher number of saccades, as well as a higher total time for the execution of the Visual Maze Test in comparison to Alcohol Condition, indicating transient motor and cognitive changes caused by the intake of alcohol. Thus, the present study aimed to investigate the effects of moderate alcohol intake in eyes movements as an indicator of cognitive processing underlying the visual search in the Maze task. In this study, we investigated the concentration of 0.08% BAC.

## Materials and methods

### Experimental design

This study used an intra-subject, double-blind, and placebo-controlled experimental design.

### Subjects

The subjects of this study were 20 volunteers (11 men and nine women), with ages ranging between 18 and 32 years (22.9 ± 6.07 years old), who consumed alcohol occasionally and in moderate quantities [1]. All volunteers participated in both conditions: experimental, after consuming alcohol (0.08% BAC), and placebo (0.00% BAC).

Regarding the inclusion criteria, the participants had to present normal monocular visual acuity (20/20) or corrected, which was evaluated through “E” Rasquin optotypes. In addition, the participants were assessed for the presence of congenital dyschromatopsia using the Ishihara pseudoisochromatic plates [36], which were binocularly applied from a distance of 75 cm. Alcohol drinking habits and possible symptoms related to alcoholism were tracked through the Alcohol Use Disorder Identification Test (AUDIT), which was previously adapted for use in Brazil by Santos, Gouveia, Fernandes, Souza, and Grangeiro [37]. In addition, the Beck Depression and Anxiety Inventories were used [38] with the goal of tracking possible symptoms related to depression and anxiety, considering a cut-off point equal to or higher than 20 points, which was used in a study with a Brazilian sample [39]. All volunteers demonstrated that they were in good physical health and were moderate and acute drinkers (2.1±1.0 times per week).

The presence of visual or neurological diseases that could affect visual functions and being pregnant were the exclusion criteria. Other participants who were excluded from the sample were individuals who consumed alcohol 24 h before the tests, exhibited any psychiatric disorder, were taking medication or other toxic substances, and presented a personal or family history (considering parents and siblings) of alcohol or illegal substance dependence. These criteria were verified through a sociodemographic questionnaire that was developed by the researchers themselves. The sample rate of eye movements recorded by the eye tracker was another exclusion criterion; participants who had less than 90% of their eye movements recorded by the equipment were excluded. Approximately 54 potential participants were contacted via telephone and e-mail. Of these, only 36 individuals participated in the screening. The main reason for exclusion were depressive symptoms (n = 2) or anxiety (n = 3), a family history of alcoholism (n = 3), and the use of illegal substances (n = 4).

### Ethics statement

This study was approved by the Research Ethics Committee of the Center of Health Sciences of the Federal University of Paraíba (Universidade Federal da Paraíba), located in the city of João Pessoa, state of Paraíba, Brazil, under protocol number 38389114.9.0000.5188, approved February 19, 2015. Participation in the research was voluntary, and all participants signed the informed consent form before the experiment was conducted, following the Code of Ethics of the World Medical Association (Declaration of Helsinki) for experiments that involve human beings.

### Equipment

Eye movements were recorded through a binocular, 300 Hz, Tobii eye tracker, attached to a 23” monitor (1920 x 1080 pixel maximum resolution and 300 cd/m^2^ max luminance) on which the Visual Maze Test (70,42 cd/m^2^ luminance) [40] was presented for the participants to visualize. The system, integrated into a Dell Latitude 3450 notebook with a 14" HD monitor (1366 x 768) and Windows 8.1 Pro 64 bit operational system, Intel Core i7-5500U 2.4 GHz processor, and 8 GB RAM memory, was used to monitor the test. In the same notebook, Tobii Studio Software, version 3.4.0, was installed; this program is a platform that allows tests to be prepared and recorded as well as output and descriptive analysis of the eye movement data. Eye movements were recorded using the protocol with an I-VT fixation filter, which categorizes eye movements based on the eye directional deviation speed. The fixation filter of Tobii Studio is an implementation of a classification algorithm proposed by Olsson [41].

### Stimulus

**Fig 1:**
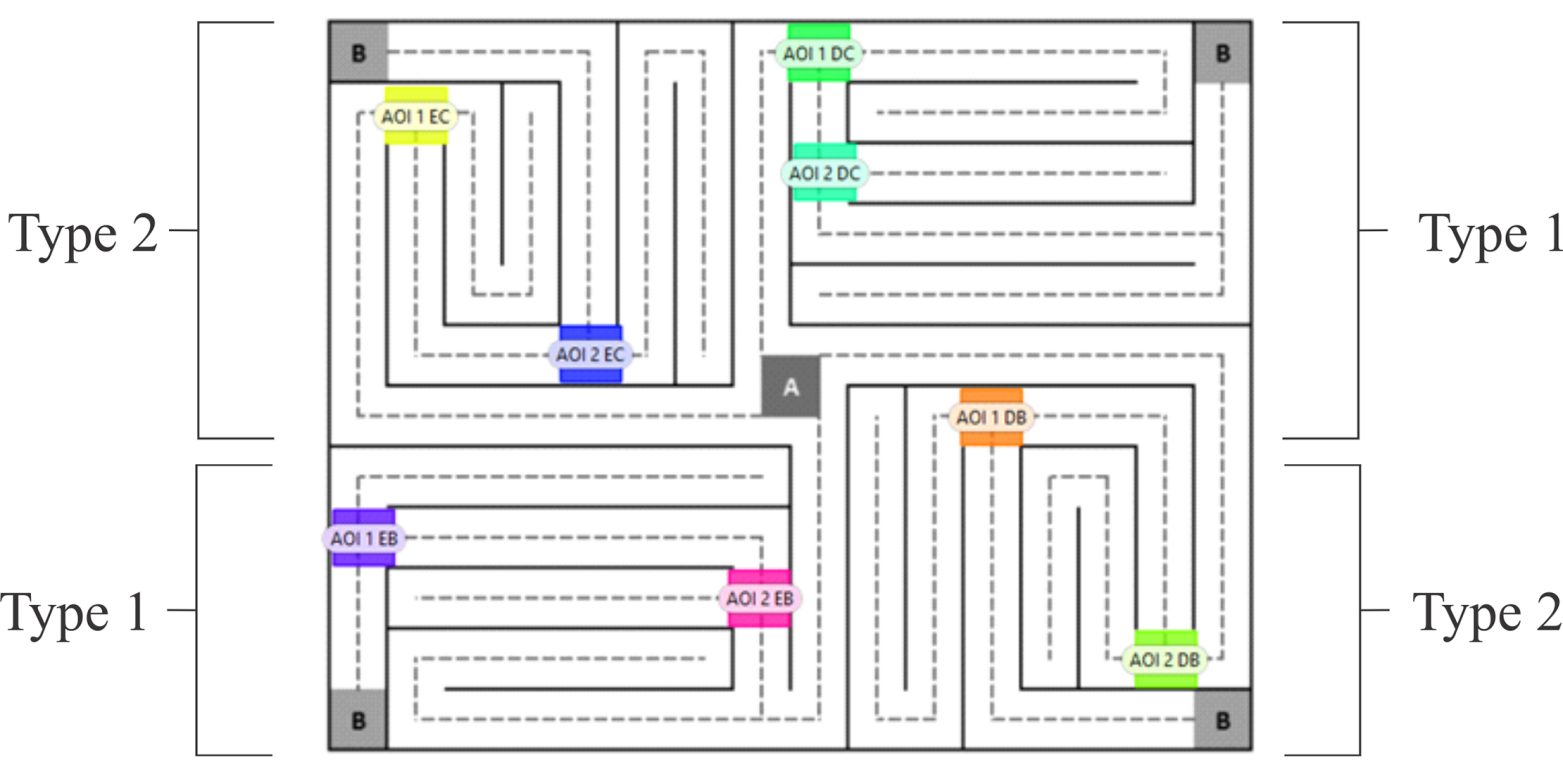
The Visual Maze Test. The rectangles are decision points (AOI’s) and the codes represent the location of the AOI's in each type of maze path.

The Visual Maze Test was used to evaluate eye movements during the search of the maze exit. This task was developed by Santos, Campos Neto, Sousa, Pessoa, and Nogueira [40] in the Perception, Neuroscience, and Behavior Laboratory of the Federal University of Paraíba. This test shows areas that, which in a brief period of time, it must be decided which path to follow (decision points). Therefore, this problem resolution task requires cognitive processing, in which processes operate such as flexibility, inhibitory control, attention, planning, visual attention and decision making, and in an integrated manner, allow the individual to guide behavior to the goals and solve problems [28]. There are two areas of interest (AOI) in the path between initial point and exit of maze, which correspond with the decision points. It is a rectangular visual maze with an initial point “A” in the center of the screen (the standard fixation initial point established by the eye tracker) and four arrival points “B,” one in each corner, with the same difficulty level, in addition to a distribution of pairs in symmetric pathways (pair Type I and pair Type II) (Fig 1). Additionally, the symmetric pairs are relevant when the participant fails in the first attempt, i.e., if the participant does not find the exit in the Type I pathway, then the researcher may subsequently instruct him/her to try through the Type II pathway. There are also dotted lines that indicate the pathway to guide the eye movements and to reduce participant anxiety. The test ended when the participant found the exit of the maze. This test is a relatively simple task, which is shown to be more resistant to psychosocial factors and individual differences. For example, educational level is often a significant factor in neuro-cognitive tests that require the recognition of numbers or letters [40].

### Procedure

The research was divided in two phases: (1) screening and (2) eye tracking evaluation. In the first phase, the participants who complied with the inclusion criteria noted above were invited to participate in the experiment. The second phase lasted for two days with an interval of 20 ± 15 days (washout phase) to prevent interference due to the order of participation in the alcohol and placebo conditions, which were determined in counterbalanced order among the subjects. The experiment was conducted individually in the afternoon between 2:00 and 6:00 P.M. Upon their arrival at the laboratory, all participants were questioned as to whether they followed the instructions to fast for 2 h. In addition, the BAC was measured before the beginning of each experimental session to confirm that the participants had not ingested alcohol 24 h prior to the experiment.

The alcohol administration consisted of vodka of the *“Wyborowa”* brand, with 40% alcohol concentration per volume, diluted in orange juice without sugar in a ratio of 1:3. In the placebo condition, the alcoholic beverage was replaced by tonic water, and two table spoons of powdered lime juice were added to the orange juice. In addition, approximately 10 ml of vodka (an insufficient amount to be recorded by the breathalyzer) were added to mask the placebo, simulating the aroma of an alcoholic beverage. In both conditions the alcoholic doses were also visually similar.

The quantity of alcoholic beverage ingested by each participant was calculated through *Dosagem* software, which was developed in the Perception, Neuroscience, and Behavior Laboratory and adapted based on the equations suggested by Brick [42] (see in [3]). This software calculates the quantity of alcohol to be ingested to achieve a BAC of 0.08%. However, the calculation for women and men, which is based on anthropometric characteristics such as weight, height, and quantity of body water, is different. The dose that each individual should consume was divided into two glasses, and the participant was given 4 min to drink each glass.

The experiment was conducted by two researchers because the present study follows a double-blind experimental protocol. Therefore, researcher #1 prepared the alcoholic beverages and measured the BAC, whereas researcher #2 administered the tests and supervised the session. Initially, researcher #1 conducted the first BAC measurement. If the BAC was 0 (which it invariably was), the participant received from researcher #1 his/her first glass of the drink. Two minutes after drinking the first glass, the participant received the second glass.

The BAC measurement in the blood was conducted at five moments, i.e., before the beverage administration and 20, 40, 60, and 90 min after the beverage administration, using the digital breathalyzer model BFD-50, which provides an indirect measure of assessing the quantity of alcohol in the blood through the air exhaled. During the placebo condition, the alcohol content in the blood was measured in the same manner as under the alcohol condition. This alcohol administration method was adapted from previous studies conducted by Silva et al., Abroms et al., and van Ravenzwaaij, Dutilh, and Wagenmakers, who have demonstrated the effectiveness of this type of placebo-controlled experimental design [3, 17, 43, 44].

**Fig 2:**
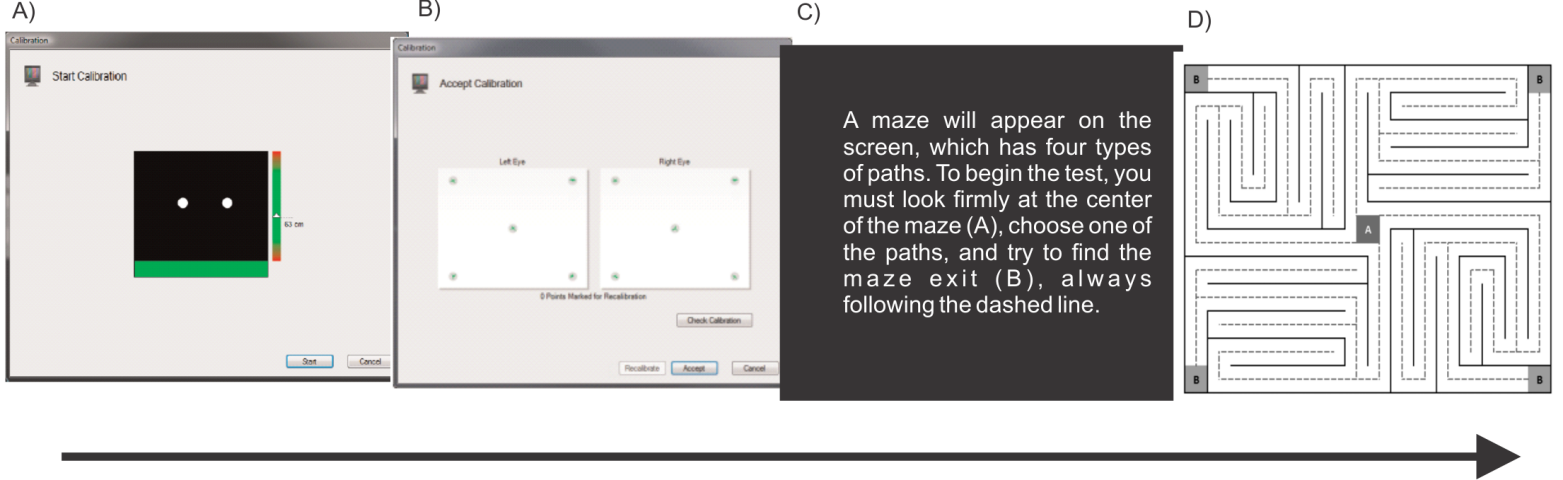
Sequence of eye tracking performance. **A)** Measurement of the distance between the eyes and the monitor screen and the beginning of the eye calibration. **B)** Calibration verification. **C)** Instructions for performing the test. **D)** Presentation of the Visual Maze Test.

The eye tracking was initiated when the BAC reached approximately 0.08%, which occurred approximately 30 min after the beginning of alcohol ingestion, for this reason, in placebo condition, the eye tracking also was initiated at this time. In the two conditions, the participants were asked to sit 65 cm from the monitor (this measure was assessed by the eye tracker itself), in a fixed position. Subsequently, a five-point calibration to synchronize the eye point, which was calculated by the eye tracker and the current eye position, was performed. Shortly thereafter, instructions were displayed on the monitor, directing the participant to fix his/her eyes on the maze’s central point (A), choose one of the pathways, and attempt to find the maze’s exit (B), continuously orienting him/herself through the dotted line. When the participant demonstrated that he/she fully understood the instructions, the maze image was displayed in the center of the screen on a white background. The sequence of the eye tracking procedure is presented in Fig 2.

Approximately 2 h after ingestion, the alcohol content in the participant’s blood was measured so that the individual would leave the laboratory with the same quantity that was recorded when he/she arrived, thus preventing any types of problems for the subjects.

### Statistical analyses

The statistical analyses were conducted using SPSS (Statistical Package for the Social Sciences), version 20. The Kolmogorov-Smirnov test showed that the data complied with the assumption of normality; therefore, parametric tests were applied. In addition, using Student’s t-test for repeated measures, significant differences between the participants’ performance in the alcohol and placebo conditions (alcohol as independent variable) in relation to the parameters of the eye tracker were observed. A 5% alpha value was considered for all statistical procedures. Additionally, Pearson bivariate correlations were performed with the goal of assessing the associations among the parameters of the eye movement, alcohol drinking habits, and the AUDIT score.

Regarding the metrics of the dependent variables analyzed in this study: the first fixation latency, the fixation and saccade duration (mean and total) variables were measured in ms, whereas the fixation and saccade number variables were measured by the frequency of their occurrence.

## Results

### Sociodemographic characteristics and alcohol consumption

The volunteers were between 18 and 32 years old (22.9 ± 6.07 years old), and the majority were undergraduate students (40%). In addition, the largest portion of the sample consisted of singles (80%), Catholics (50%), those with a monthly income three to five times the minimum wage (55%), and those who presented right-eye dominance (65%). All participants were exhibited normal (65%) or corrected visual acuity (35%). Regarding alcohol drinking habits, the majority had been drinking for 10 (30%) and five (20%) years, and beer was their beverage of choice (40%). Table 1 describes the alcohol drinking habits and the AUDIT score, which were grouped by gender. These characteristics did not present significant differences between men and women.

**Table 1:** Alcohol drinking habits and AUDIT score.

	**Men *(n = 11)***		**Women *(n = 9)***	
	*M*	*SD*	*M*	*SD*
**Consumption time period (years)**	6.7	3.1	5	2.9
**Frequency (week)**	2.1	1.7	1	0.0
**Number of doses (cups) per occasion**	7	4.4	4.2	2.6
**AUDIT**	10.2	3.9	6.8	3.3

### Blood alcohol concentration

**Fig 3:**
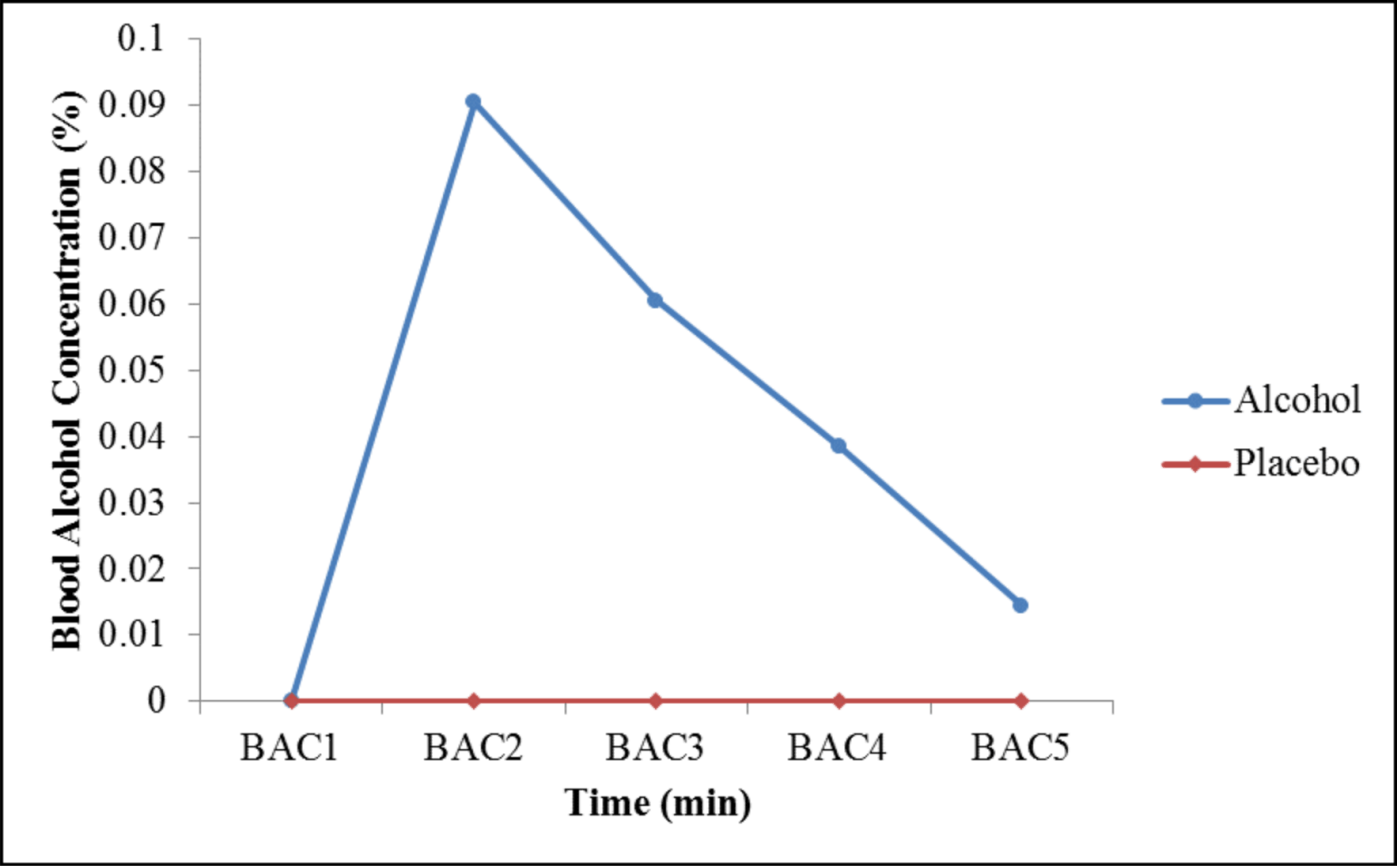
Blood alcohol concentration (BAC) in the alcohol and placebo conditions.

Fig 3 shows the mean alcohol concentration at each time interval that was analyzed. In the alcohol condition, the mean BAC 20 min after the alcohol administration (BAC2) was 0.09% (SD = 0.018). Approximately 40 min after ingesting the alcohol (BAC3), the mean was 0.06% (SD = 0.017). The BAC means at 60 and 90 min were 0.03% (SD = 0.016) and 0.014% (SD = 0.010), respectively. The peak BAC occurred approximately 20 min after the dose was completely ingested.

Regarding the BAC differences between men and women, men presented a higher alcohol concentration at BAC2 (0.10 ± 0.01) than women (0.07 ± 0.01), which was a significant difference [*t*(18) = 3.52, *p =* 0.01]. At BAC3, there was a significant difference [*t*(18) = 2.14, *p =* 0.46] between men (0.06 ± 0.01) and women (0.05 ± 0.02). However, there were no significant differences between the genders at BAC4 and BAC5. In addition, after 90 min, the BAC decreased to 0.014%. As expected, in the placebo condition, the breathalyzer did not detect alcohol concentrations in the subjects’ blood (0.00%).

### Eye movement parameters

**Fig 4:**
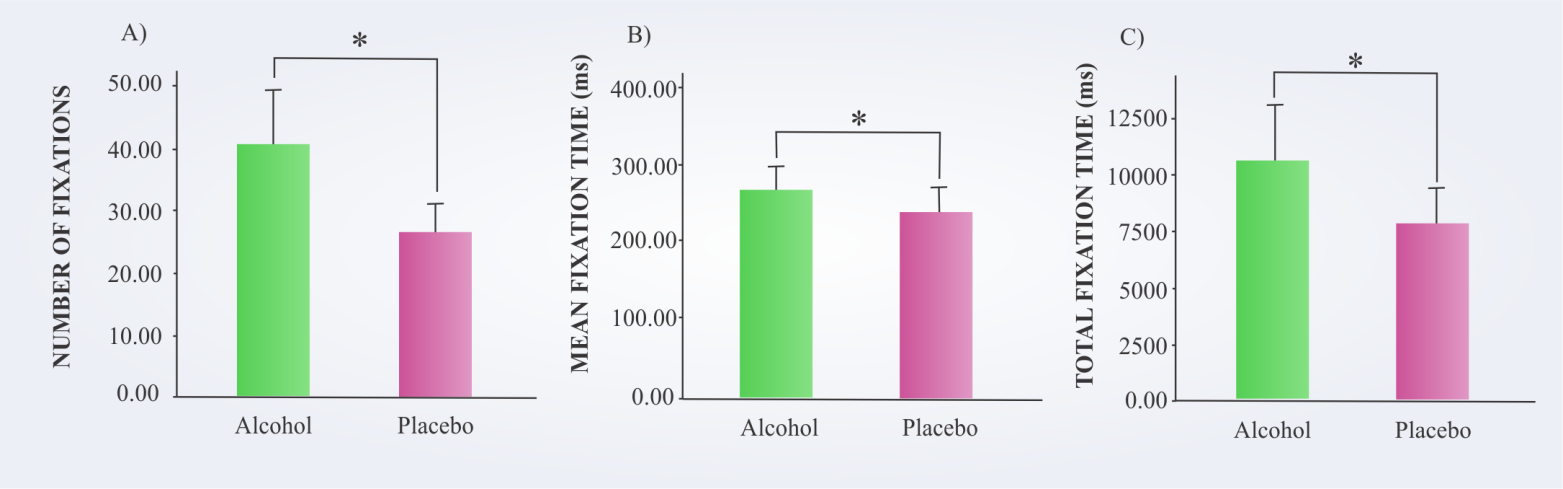
Participants performance in the alcohol and placebo conditions. **A)** Number of fixations. **B)** Mean fixation time. **C)** Total fixation time. *p < 0.05.

Fig 4 compares the subjects’ performance in both conditions regarding the fixation parameters. In the alcohol condition, the subjects exhibited a significantly larger total number of fixations [*t*(19) = 4.434, *p =* 0.001, *r* = 0.713], mean fixation time [*t*(19) = 3.245, *p =* 0.004, *r* = 0.597], and total fixation time [*t*(19) = 2.418, *p =* 0.026, *r* = 0.485] compared to the performance in the placebo condition.

**Fig 5:**
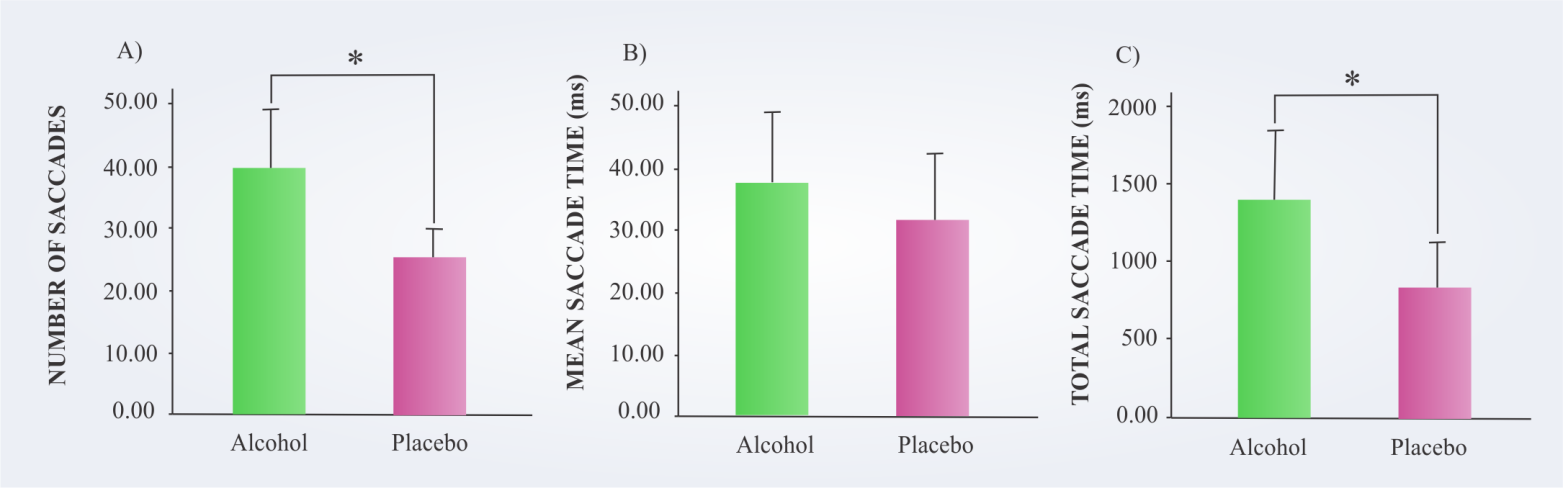
Saccadic movements of the subjects in the alcohol and placebo conditions. **A)** Number of saccades. **B)** Mean saccade time. **C)** Total saccade time. *p < 0.05.

Fig 5 compares the subjects’ performance in both conditions regarding the saccade parameters. In the alcohol condition, the participants executed a significantly higher number of saccades [*t*(19) = 4.633, *p =* 0.001, *r* = 0.728] and a higher total saccade time [*t*(19) = 2.374, *p =* 0.028, *r* = 0.478] in relation to the placebo condition. However, there was no significant difference for the mean saccade time between the conditions [*t*(19) = 0.838, *p =* 0.413, *r* = 0.188].

Table 2 summarizes the descriptive statistics related to all the eye movement parameters that were analyzed during the execution of the task. It is observed that the first fixation latency was significantly higher in alcohol condition [*t*(19) = 2.806, *p* = 0.011, *r* = 0.541] than in the placebo condition, i.e., participants provided alcohol showed reaction time increased to begin the test. The task execution time was higher in the alcohol condition [*t*(19) = 2.695, *p =* 0.014, *r =* 0.525] than in the placebo condition.

**Table 2:** Descriptive statistics for the eye movement parameters in the alcohol and placebo conditions (n = 20).

	Alcohol Condition		Placebo Condition	
	*M*	*SD*	*M*	*SD*
First fixation latency (ms)	62.00	122.4	32.25	81.82
Number of Fixations	40.60	19.30	26.55	10.11
Mean fixation time (ms)	272.6	60.40	246.5	55.65
Total fixation time (ms)	10488.2	5545.5	7758.7	3482.7
Number of saccades	40.15	19.61	25.45	10.26
Mean saccade time (ms)	37.75	23.87	31.61	23.01
Total saccade time (ms)	1410.25	932.27	838.70	602.90
Task execution time (ms)	11355.05	5353.76	8525.60	3561.13

**Fig 6:**
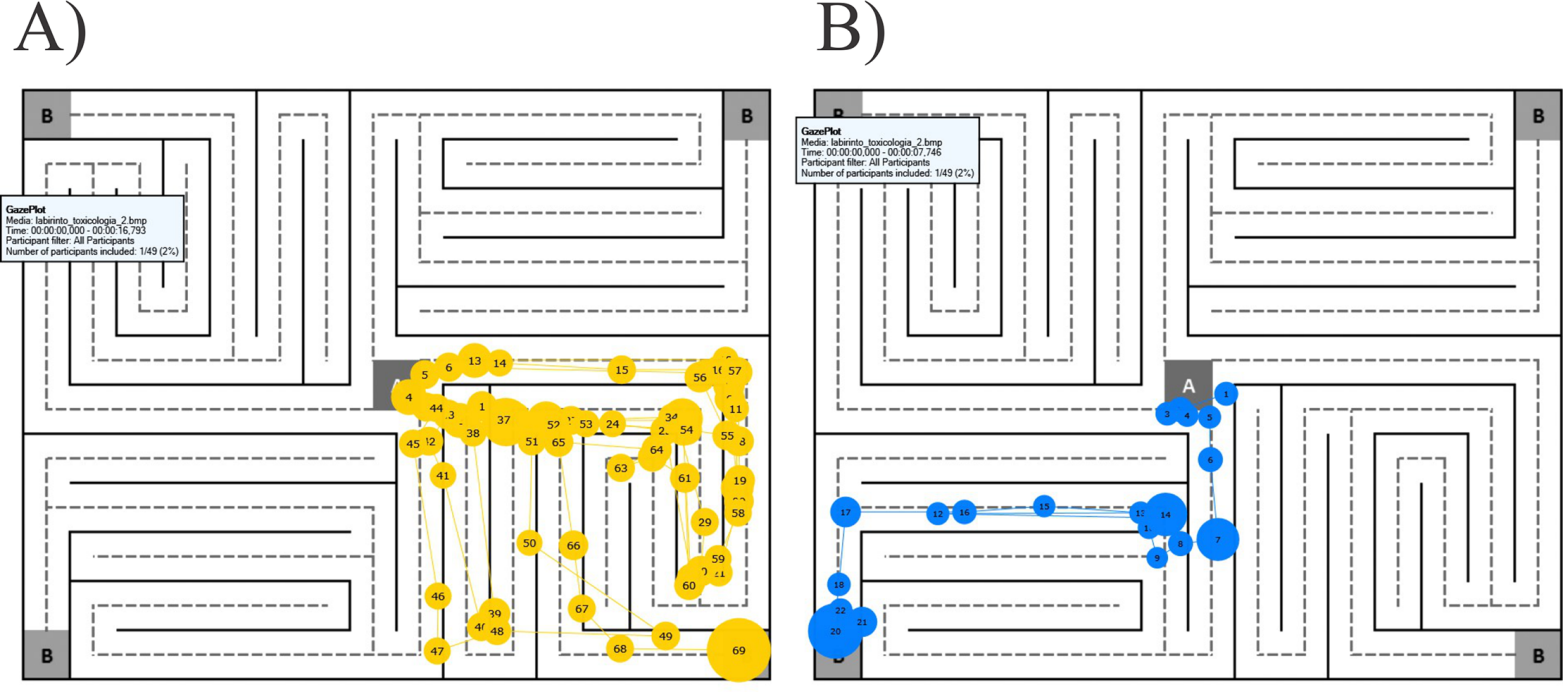
Gaze plots representing the fixations and saccades. Performance of Subject A01014 (male, 22 years old) in both experimental conditions. **A)** Eye movements obtained from the alcohol condition. **B)** Eye movements obtained from the placebo condition. Circles depict fixations and their diameters indicate duration of each fixation. Numbers within the circles represent the fixation sequence. Lines connecting fixations represent the saccades sequence.

The areas of interest (AOIs 1 and 2) were analyzed, which are the points at which the participant must decide which path to follow to find the way out of the labyrinth. It was observed that the participants on the condition alcohol had a significantly greater number of fixations in both AOI 1 [*t*(19) = 5.616, *p* = 0.001, *r* = 0.789] as on the AOI 2 [*t*(19) = 5.085, *p* = 0.001, *r* = 0.759], in comparison to the placebo condition. Fig 6 shows the gaze plots that represent the participant’s eye movements in the alcohol and placebo conditions. The gaze plots portray the basic components of eye movements, fixations and saccades. In the alcohol condition, the participant performed a higher number of fixations and saccades in finding the maze’s exit than in the placebo condition.

In addition, we analyzed the existence of correlations between the parameters of ocular movement and alcohol drinking habits. In alcohol condition, the Pearson correlation coefficient showed that the number of fixations positively correlated with the number of doses consumed per occasion (*r* = 0.736, *p =* 0.001) and AUDIT score (*r* = 0.703, *p =* 0.002). The total fixation time also showed a positive correlation with the number of doses ingested per occasion (*r* = 0.479, *p =* 0.038) and AUDIT score (*r* = 0.549, *p =* 0.022). In placebo condition, the number of saccades positively correlated with the number of doses consumed per occasion (*r* = 0.730, *p =* 0.001) and AUDIT score (*r* = 0.691, *p =* 0.002).

## Discussion

The consumption of alcoholic beverages is observed in almost all cultures, and the negative impacts of alcohol on public health are related to chronic use and moderate acute consumption [1, 45]. The alcohol works as a depressor of the CNS [7, 12], which may alter diffusely cognitive and perceptual processes [2, 6, 18]. Therefore, the present study examined the effects of moderate acute alcohol ingestion (0.08% BAC) on the eye movement pattern, as an indicator of cognitive processing [34].

Based on Xiao and Ye, alcohol consumption may cause a potential decrease in the activity of GABA, the main inhibitory neurotransmitter in the brain, which is found in the retinal ganglion and bipolar cells, the LGN, the SC and the visual cortex [12], involved in the processing of visual functions, including the ocular movement control [46], as well as affects other neural structures related to cognitive processes such as attention, inhibitory control, reaction time, decision making [47].

We predicted that young adults under the influence of a moderate dose of alcohol may present subclinical alterations in their eye movements, indicating impairment in cognitive processing. The results confirmed our hypothesis: participants in alcohol condition showed a longer first fixation latency and a higher fixation number and duration (mean and total) in the Visual Maze Test compared to the placebo condition. Such findings support the idea that the moderate acute alcohol consumption may lead to alterations in the eye movements pattern. Based on Goldberg and Kotval, eye movements are intrinsically related to cognitive processing because a higher number and time of fixations negatively correlate with the efficiency of the visual search. In addition, the less time to first fixation, may indicate impaired reaction time and attention orientation [48]. In this sense, it is suggested that participants under the influence of alcohol showed less efficiency in cognitive processing during the resolution of the problem proposed in the visual maze test.

In alcohol condition, participants also showed a greater number and duration of saccades and needed more time for the execution of the Visual Maze test, in comparison to placebo condition. In line with Hoffman, the saccadic movements are closely linked to the visual attention, suggesting that attention is oriented in the same direction of saccades [23]. Thus, these results suggest that alcohol can affect the attentional control and reduce the accuracy of location visual targets [5], i.e., the individuals under the effect of alcohol need to perform a greater sweep of sequential elements to find the way out of the maze [16, 49]. Thus, these effects found may suggest effects of alcohol on the top-down components of visual attention, considering that the eye movements are influenced by top-down processes, that require cognitive activity in order to set the goal oriented behaviors [25], as in the case of the Visual maze test.

Our results corroborate studies that suggest that moderate alcohol ingestion may affect eye movements, in which this alterations evidence impairments in top–down regulative cognitive functions [4, 17, 18, 50]. Although both studies have within subject design, a direct comparison between our findings and those of other studies that assess the relationship between alcohol use and eye movements is difficult due to methodological differences related to, for instance, the form in which the dose of alcohol is administered, the eye tracker equipment, the sample size, and the eye movement parameters that were analyzed. For instance, this is the case for the study by Marinkovic et al. [5]; although these authors not used double-blind design and administer different doses among participants based on sex (0.6 g/kg of alcohol to male participants and 0.55 g/kg to female participants) different from that used in the present study, in which we use software that is based on anthropometric characteristics to measure the exact amount of alcohol to be ingested to achieve 0.08% BAC. Moreover, our work differs from other studies [5, 17, 18] by the visual stimulus used, which is a complex task that involves problem solving and decision making.

Regarding the study of Abroms et al. [17], for example, our study differs in relation also to the task and the dosage administered. The authors manage the alcoholic dosage containing a part of alcohol and three parts of mixing carbonated. However, both are similar in a blinded experiment. Despite of these differences, our findings confirm the results found in the literature, indicating that an alcoholic beverage is able to change the eye movements and cognition aspects [4, 16, 18]. It should be emphasized that the studies reported [5, 17] provided methodological support for this work, and the methodological improvements made sought to control the largest possible number of variables involved, providing greater robustness to the results.

In addition, the test used in this study presents areas of decision in which the individual must decide which path to follow to find the way out of the maze. According to Krajbich et al., the evidence that assist in decision are accumulated during the fixations [3]. Our findings showed that that the participants on the alcohol condition had a significantly greater number of fixations in both areas of interest, in comparison to the placebo condition. In this sense, changes in eye movements during the resolution of the task give evidence of the influence of this substance in decision-making and, consequently, in other processes involved in the ability to choose, such as inhibitory control, planning, and visual attention, which in an integrated way, allow the individual to guide behavior regarding goals and solve problems [28], such as the resolution of the maze task. From this results, the need for a greater number of fixations may indicate difficulties in decision-making process during a visual search [3, 16].

Additionally, the correlations that were evaluated in this study show that alcohol drinking habits positively correlate with the number and duration of fixations and saccades, which suggests that individuals who ingest a larger number of doses per occasion perform more fixations and saccades when executing the Visual Maze Test. The same is found for the subjects who present a high AUDIT score, i.e., individuals who consume alcohol more frequently and ingest a larger number of doses per occasion must perform more fixations and saccades in executing the test, which suggests possible long-term effects of alcohol consumption [45] on perceptual and cognitive processes.

Nevertheless, some considerations must be presented regarding the alcohol drinking habits in the sample studied. Although the sample consists of social drinkers, the mean AUDIT score shows harmful patterns of alcohol use among the male participants (score > 10). These scores may have been obtained because the sample mostly consists of college students, who frequently inflate the score in the instrument because, in this population, social events are common and alcohol use is encouraged. Similar scores have been found in other studies that assess the effects of alcohol and use samples that are similar to those used in the present study [51, 52].

In general, several studies have shown impairments in different motor, visual and cognitive functions associated with acute alcohol consumption [16, 17, 18]. On the other hand, there are results that suggest beneficial effects of alcohol on cognition [53] which is the case of the study carried out by Britton, Singh-Manoux and Marmot [54]. These authors investigated the relationship between alcohol consumption and cognitive function (they assessed memory, verbal and mathematician reasoning, recognition and understanding of semantic fluency words and phonemic awareness) and they concluded that for middle-aged individuals, alcohol improves some cognition aspects. Moreover, alcohol consumption can have a protective effect against coronary disease and, possibly, an ischemic stroke, however, these results should be interpreted with caution, since the benefits of alcohol consumption may be outweighed by the other diseases (including liver cirrhosis and Alcoholic psychoses), violence and traffic accidents [54].

Studies on eye movements are relevant because they provide strong links to cognitive processing [55]. However, the investigation of eye movements have been challenging due to wide range of eye tracking protocols, visual stimuli and eye movement parameters analyzed, which hinders comparisons among studies. In the literature on this theme, for instance, no studies on eye tracking that use a visual task similar to the visual maze used in this study are found. Considering the results obtained, the Visual Maze Test demonstrates that it is an efficient task for evaluating visual information processing, resistant regarding psychosocial and individual factors, and is in line with a previous study in which this test is described [40]. However, additional studies that use this test are needed.

In summary, our results confirm that moderate doses of alcohol can change the eye movements of young adults, giving evidence of the influence of alcohol in cognitive processes, such as planning, visual attention and decision-making, which operate in an integrated manner in the resolution of the Maze Task. Thus, findings of this study add a significant step in understanding the diffuse transitory impairments caused by alcohol in perceptual and cognitive processes analyzed from deficits in the pattern of eye movements. Moreover, assessment of eye movements is relevant due to the essential role of eye movements in performing several everyday activities such as traffic mobility and spatial orientation. Therefore, considering the effect of alcohol consumption on public health, studies that focus on describing possible perceptual and cognitive alterations in adults who are exposed to moderate alcohol use are relevant by encourage public policies that prioritize preventive measures against health damage that is the result of the use of alcohol.

## Supporting information

S1 Dataset Data of eye movement parameters for alcohol and placebo conditions. Eye movements parameters of young adults in the Visual Maze Test in both conditions.
(XLSX)

S2 Dataset Data of blood alcohol concentration (BAC) levels for alcohol and placebo conditions. (XLSX)
